# Leader Humor and Employee Job Crafting: The Role of Employee-Perceived Organizational Support and Work Engagement

**DOI:** 10.3389/fpsyg.2020.499849

**Published:** 2020-10-08

**Authors:** Ling Tan, Yongli Wang, Wenjing Qian, Hailing Lu

**Affiliations:** ^1^School of Management, Guangdong University of Technology, Guangzhou, China; ^2^Business School, Sun Yat-sen University, Guangzhou, China; ^3^School of Economics and Management, Nanjing University of Science and Technology, Nanjing, China

**Keywords:** leader humor, job crafting, work engagement, perceived organizational support, JD-R model

## Abstract

Research on the outcomes of leader humor has mainly focused on attitudinal or in-role behaviors, while proactive change-oriented behaviors have been neglected. Addressing these issues is important for scholars and practitioners to better understand how leader humor enables subordinates to behave proactively. By integrating the resource accumulation perspective and the motivational process of the Job Demands–Resources (JD-R) model, we frame leader humor as a socioemotional resource that can help employees to create other forms of resources, such as job resources (i.e., perceived organizational support). In turn, these job resources relate to employees’ motivations (i.e., work engagement) and behaviors (i.e., job crafting). We predict that leader humor is positively related to seeking resources and challenges and negatively associated with reducing demands through the serial mediating effects of followers’ perceived organizational support and work engagement. We test these hypotheses using an experimental design with a field sample in Study 1. Furthermore, we strengthen our hypotheses by replicating our results through a multiwave field study in Study 2. We consistently find: (1) a positive association between leader humor and followers’ perceived organizational support, (2) a positive link between followers’ perceived organizational support and work engagement, and (3) serial mediating effects of followers’ perceived organizational support and work engagement on the leader humor–job crafting link. The implications of the findings and future directions for research investigating leader humor and job crafting are discussed.

## Introduction

Leader humor can strengthen followers’ job performance and mental health and improve organizational effectiveness (for meta-analyses, see Mesmer-magnus et al., 2012; Kong et al., 2019). Leader humor is defined as a behavior enacted by a leader and directed toward a subordinate that is appraised by the subordinate as funny or causes the subordinate to experience amusement (McGraw and Warren, 2010; Cooper et al., 2018). Research has shown that leader humor has a broad range of consequences for employees, including improved job performance (Arendt, 2009), informal learning (Tremblay and Gibson, 2015), creativity (e.g., Huang et al., 2015), job satisfaction (Vecchio et al., 2009), affective commitment (Hughes and Avey, 2009), work engagement (Yam et al., 2018), leader–member exchange (Robert et al., 2015; Pundt and Venz, 2017; Cooper et al., 2018), and trust in leaders (e.g., Hughes and Avey, 2009).

Despite these promising findings, research on the implications of leader humor has mainly focused on attitudinal or in-role behaviors and neglected proactive change-oriented behaviors, such as job crafting behaviors, which are salient to organizational adaptation and survival (Bruning and Campion, 2018). In this vein, opportunities abound to enrich our understanding of the outcomes of leader humor at work. By examining whether and how leader humor relates to job crafting behaviors, our study answers calls from scholars to examine the positive effects of leader humor (Cooper, 2008) and to explore the factors that facilitate followers’ job crafting behavior (Li et al., 2013). Increasing job resources that stimulate work engagement and job crafting might not be easy or feasible, e.g., increasing every employee’s job autonomy, or can be costly, e.g., providing more learning opportunities. We argue that leader humor and positive behaviors are feasible and less costly. Therefore, leader humor can be a good alternative to structural job resources, especially when there is a paucity of such resources. Only by understanding the effect of leader humor behavior on employee job crafting can scholars and practitioners better understand why and how job crafting occurs and the proactive, change-oriented behavioral implications of leader humor. Thus, it is advantageous to examine whether leader humor relates to job crafting among subordinates and, if so, why and when these beneficial impacts are likely to occur.

To address these issues, we adopt a resource accumulation perspective to explore the proactive, change-oriented behavioral implications of leader humor. Specifically, as resources tend to accumulate, we suggest that the socioemotional resources (e.g., a signal of their organization caring about their well-being and valuing their contributions) elicited by leader humor can help employees to increase other resources to achieve their goals. In other words, the resources engendered by leader humor are beneficial for employees to create other forms of resources, such as job resources, i.e., perceived organizational support. Perceived organizational support is particularly relevant because supervisors act as agents of the organization, and subordinates tend to attribute the caring and support offered by their leaders to the organization itself (Eisenberger et al., 1986). To further understand the downstream outcomes of job resources offered by leader humor, we focus on the motivational process of the Job Demands–Resources (JD-R) model (Schaufeli and Bakker, 2004; Schaufeli et al., 2009). This model is very critical for understanding the associations between job resources and motivations (i.e., work engagement), as well as the behaviors (i.e., job crafting) of followers (Rich et al., 2010; Christian et al., 2011). According to the JD-R model (Schaufeli et al., 2009), high resources relate to increased motivation and greater productivity (the motivational process). By integrating the resource accumulation perspective and the motivational process of the JD-R model, we propose a sequential mediation model that specifies why leader humor relates to job crafting *via* employee-perceived organizational support and work engagement. That is, job resources (i.e., perceived organizational support) engendered by leader humor relate to work engagement, which in turn exhibits a positive correlation with job crafting behaviors.

This study makes several contributions to the literature. First, it contributes to the promising literature related to both job crafting and leader humor. Regarding the literature related to job crafting, researchers have focused on either individual factors (Bakker et al., 2012; Bipp and Demerouti, 2015; Tims et al., 2016) or job characteristics as predictors of job crafting (Petrou et al., 2012). However, research linking leader behavior to employee job crafting has been limited. Regarding the literature related to leader humor, to the best of our knowledge, the current research is the first study to empirically investigate the link between leader humor and employees’ job crafting behaviors. Accordingly, this study sheds new light on how leaders enable subordinates to behave proactively. Moreover, job crafting enables employees to better meet organizational goals by constantly initiating changes in the workplace environment (Petrou et al., 2018). Thus, exploring the effect of leader humor on facilitating employee job crafting is essential.

This study makes secondary contributions to the literature related to organizational support theory and work engagement by focusing on leader humor as a determinant of perceived organizational support. Relatively limited research has examined the relationship between leader behavior and perceived organizational support (Rhoades and Eisenberger, 2002). Moreover, this study contributes to the literature related to work engagement by examining leader humor as a potential predictor of employee work engagement. Although humor has often been theoretically linked to work engagement, empirical research in this domain has focused on humor from an individual, intrapersonal perspective, claiming that humor is shaped by individual differences rather than behaviors; accordingly, it has examined the intrapersonal outcomes of humor (Duncan, 1982; Avolio et al., 1999; Decker and Rotondo, 2001; Robert et al., 2015). To extend the previous research and theory, we frame humor as a behavior rather than a trait-like individual disposition. From this behavioral perspective, we propose that employee exposure to leader humor behaviors makes employees more likely to perceive their organization as supportive and subsequently results in a positive association with their motivations (i.e., work engagement) and behaviors (i.e., job crafting).

Moreover, the present study contributes to the literature related to leader humor and employees’ proactive, change-oriented behavior by directly testing the underlying mechanisms relating leader humor to job crafting. By integrating the resource accumulation perspective with the JD-R model, we propose that the effects of leader humor on job crafting are explained by the serial mediating effects of perceived organizational support and work engagement. First, we frame leader humor as a socioemotional resource that can be useful for employees in creating other forms of resources, such as perceived organizational support. Moreover, employee job resources and motivational states likely serve as key mediators between leader behavior and employees’ behavioral reactions (Parker et al., 2006) since the JD-R model suggests that work engagement mediates the link between job resources and organizational outcomes (Schaufeli et al., 2009). Therefore, we construct and empirically examine a serial mediation model that specifies why leader humor relates to job crafting *via* employee-perceived organizational support and work engagement.

## Theory and Hypotheses

### The JD-R Model and Leader Humor

Job resources refer to “those physical, social, or organizational aspects of the job that may do any of the following: (a) be functional in achieving work goals; (b) reduce job demands and the associated physiological and psychological costs; (c) stimulate personal growth and development” (Demerouti et al., 2001, p. 501). An example of job resources is perceived organizational support. The JD-R model suggests that work environments, events, or behaviors that provide job resources elicit a fulfilling, positive work-related state of mind (i.e., work engagement), either by satisfying a basic need or by achieving work goals. Subsequently, this affective-motivational state engenders positive outcomes, such as job crafting. Consistent with the motivational process perspective, our study investigates how leaders’ humorous behaviors enable individuals to gain job resources (i.e., perceived organizational support) at work and how these job resources in turn relate to high levels of employee work engagement, consequently enabling employees to craft their jobs (Rich et al., 2010). In other words, the JD-R model emphasizes how job resources (i.e., perceived organizational support) resulting from work behaviors (i.e., leader humor) have downstream effects on not only the motivations (i.e., work engagement) but also the behaviors (i.e., job crafting) of their followers (Rich et al., 2010).

### Leader Humor and Perceived Organizational Support

Perceived organizational support refers to “the degree to which employees believe that their organization values their contributions and cares about their well-being” (Eisenberger et al., 1986, p. 501). According to the JD-R model, work environments, events, or behaviors offer job resources. When leaders display humor, followers tend to perceive that the organization is positively oriented toward them. As a socioemotional resource, leader humor signals leader support and affability, thereby fulfilling the socioemotional needs of their employees (Cooper et al., 2018). Importantly, subordinates tend to attribute the caring and support offered by their leaders to the organization itself (Eisenberger et al., 1986) since supervisors act as agents of the organization. In this way, leader humor positively relates to the employees’ perception that the organization is supportive. Prior studies have provided support for this hypothesis. For example, a meta-analytic review demonstrated that leader behavior is associated with perceived organizational support (Kurtessis et al., 2017). More precisely, supervisor support (ρ = 0.60) and leader consideration (ρ = 0.46) were positively associated with perceived organizational support, whereas hostile supervisor behaviors (i.e., abusive supervision; ρ = −0.34) were negatively related to perceived organizational support. Thus, consistent with the theoretical arguments and empirical evidence, we propose that leader humor is related to employee-perceived organizational support.

Hypothesis 1: Leader humor is positively associated with the organizational support perceived by employees.

### Perceived Organizational Support and Work Engagement

Work engagement refers to “a positive fulfilling, affective, motivational state of work-related well-being, described by vigor, dedication, and absorption” (Schaufeli et al., 2002, p. 74). The JD-R model emphasizes the inherently motivational qualities of job resources. As employees perceive that the organization values their contributions and cares about their well-beings, they are more likely to be motivated to perform better and accomplish more. More precisely, to feel energetic toward, dedicated to, and immersed in their work, the intrinsic and extrinsic motivations of the employees must be increased (Kahn, 1990; May et al., 2004). Furthermore, high levels of perceived organizational support resulting from leader humor tend to foster employees’ intrinsic and extrinsic interests in their work, in turn inspiring them to fully invest their energy, enthusiasm, and absorption in their work. Consistent with this theoretical argument, empirical studies have discovered that follower work engagement is positively correlated with employees’ perception of organizational support (Eisenberger and Stinglhamber, 2011; Caesens et al., 2014, 2016). For example, Caesens et al. (2014) determined that perceived organizational support has a positive association with employee work engagement. A prior study revealed that perceived organizational support is positively related to the three dimensions of employee work engagement, namely, vigor, dedication, and absorption (Caesens et al., 2016). Thus, consistent with previous theoretical and empirical works, we formulate the following hypothesis:

Hypothesis 2: The organizational support perceived by employees is positively associated with their work engagement.

### Work Engagement and Subordinate Job Crafting

Beyond the positive relationship between perceived organizational support and work engagement (e.g., Vogelgesang et al., 2013), there is also a strong association between work engagement and job crafting. Job crafting is defined as self-initiated job design behavior aiming to satisfy employees’ needs and goals by seeking resources, seeking challenges, and reducing demands (Tims et al., 2012). Job crafting behaviors can involve developing knowledge and skills, performing challenging tasks, and avoiding overly demanding tasks. Since the JD-R model suggests that work engagement fosters positive employee outcomes, we propose that employees with high levels of work engagement will engage in seeking resources and challenges but not in reducing demands. Referring to seeking resources and challenges, highly engaged employees care about and value their work and thus are expected to invest additional effort to improve their work situations (Hallberg and Schaufeli, 2006). This extra effort is likely to stimulate employee resource-seeking behavior, such as seeking learning opportunities and assuming additional responsibilities. Furthermore, employees with high levels of vigor and energy are more likely to seek challenges, such as fulfilling additional responsibilities, because high engagement can supply employees with the energy and dedication necessary to engage in seeking challenges (Sonnentag, 2003). In addition, highly engaged employees tend to express optimism, passion, and enthusiasm at work (Den Hartog and Belschak, 2012). These positive emotions are likely to expand employees’ thought–action repertoire by improving their cognitive abilities such that they are more creative and better able to achieve personal growth and meaningful performance (Bindl et al., 2018). In contrast, referring to reducing demands, we expect that employee work engagement is negatively associated with reducing demands. Highly engaged employees will not decrease their workload because the optimal level of the challenge of the job is an integral part of work engagement. In other words, by decreasing their workloads, employees unwittingly also decrease the triggers of or need for action. Indeed, empirical studies have indicated that work engagement is positively related to seeking resources and challenges and negatively associated with reducing demands (Rudolph et al., 2017; Lichtenthaler and Fischbach, 2019; Zhang and Parker, 2019). Therefore, we test the following hypotheses:

Hypothesis 3: Work engagement is positively associated with seeking resources (3a) and seeking challenges (3b) but negatively associated with reducing demands (3c).

**FIGURE 1:**
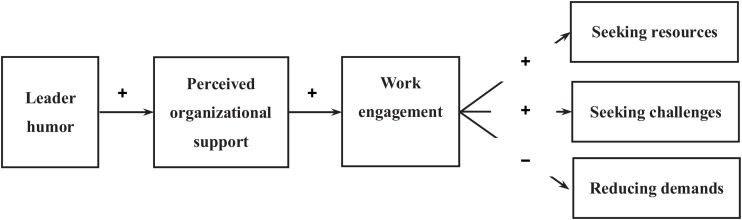
Theoretical model.

Based on the arguments above, we create a serial mediation model of the effects of leader humor on employee job crafting behaviors to elaborate on the proactive, change-oriented behavioral implications of leader humor. Leader humor is a socioemotional resource that people obtain and develop to attain additional benefits (Cooper, 2008), such as job resources (i.e., perceived organizational support). In turn, these resources are prone to impacting not only the motivations (i.e., work engagement) but also the intentions (i.e., job crafting intention) and behaviors (i.e., job crafting) of their followers (Rich et al., 2010). Please note that, although intentions and behaviors do not refer to the same construct, generally behaviors and intentions are linked. In Study 2, we use Petrou’s scale to assess past job crafting behaviors, whereas in Study 1, we argue that activation of the notion that a leader is humorous can lead to an intention to craft more frequently in the future. Thus, we changed the framing of the items on the questionnaire for Study 1 (i.e., “I will probably ask for more responsibilities”). Therefore, by integrating a resource accumulation perspective with the JD-R model, we propose that leader humor facilitates employees’ acquisition of job resources (i.e., perceived organizational support). This outcome, then, subsequently leads to high levels of work engagement, motivating employees to engage in job crafting behaviors. Accordingly, we posit the following hypothesis with respect to the downstream effects of leader humor on employee job crafting behaviors:

Hypothesis 4: Employee-perceived organizational support and work engagement mediate the effects of leader humor on employee job crafting in the form of seeking resources (4a), seeking challenges (4b), and reducing demands (4c). Figure 1 presents the theoretical model of the hypothesized relationships.

### Ethics Statement

The study was conducted in accordance with the guidelines established by the Declaration of Helsinki and in compliance with the APA ethical guidelines. Written consent was obtained from all of the participants. Anonymity and confidentiality were assured. The participants could freely withdraw at any time.

## Study 1

To test the theoretical model of why and when leader humor enhances employee job crafting, we conducted two studies of full-time employees. One had an experimental design, and the other was a multiwave field study. In the experimental study, we examined our hypotheses to ensure internal validity *via* an experimental–causal-chain design. In the second study, we cross-validated the results of the first study using a field survey method to ensure external validity.

### Sample and Procedures

In total, 214 full-time employees were recruited through the alumni networks of a few universities in China. Of these participants, four were excluded from the analysis because they had not experienced any of the incidents described in the survey. The final sample consisted of 210 participants (45.2% male, 76.2% married) who were randomly assigned to one of the following two conditions, specifically low humor (*N* = 106) or high humor (*N* = 104). The majority of the participants were aged between 20 and 40 years old, thus accounting for 92.4% of the participants, whereas participants between 41 and 50 years old accounted for 6.2%, and those 51 years old and older accounted for 1.4%. Participants with an organizational tenure of less than 5 years and those with between 5 and 10 years accounted for 39 and 40.5%, respectively, whereas those with 10–15 years and those with 16 years or more accounted for 14.3 and 6.2%, respectively. With respect to education, approximately 66% had received a master’s degree or higher, 76.7% had a bachelor’s degree, and the remaining participants, approximately 23%, had a community college degree.

This study used the critical incident technique (Flanagan, 1954), which has been widely used in prior works (Liang et al., 2016; Wellman et al., 2016; Wang et al., 2018). As a qualitative research method, the critical incident technique is used to obtain in-depth knowledge about and understanding of subjects’ responses to selected situations. This approach facilitates the investigation of significant occurrences, which could be events, incidents, processes, or issues identified by the respondent. In addition, it enables the researcher to explore what the incident is about and why it is perceived to be significant, how it was managed, and what its perceived consequences were. The objective is to gain an understanding of the incident from the perspective of the individual, considering his or her beliefs, feelings, and actions (Gremler, 2015). Online questionnaire design software^1^ was used to create the questionnaire link, which consisted of both a high-humor condition and a low-humor condition version. This software shuffled the two versions every time that the participants clicked the questionnaire link to ensure that all of the participants were randomly assigned to one of the two versions, i.e., high-humor condition or low-humor condition. We followed previous studies’ manipulations to develop the high- vs. low-humor conditions (e.g., Bitterly and Schweitzer, 2019; Ju et al., 2019). The participants were required to recall an incident in which the leader communicated with them either in a humorous manner (“Please recall a particular incident in which the leader communicated with you in a fun, humorous manner at work within the last 3 months. This leader expressed humor with you at work. For example, this leader shared humorous events or stories with you at work; this leader spread humor into many types of situations when interacting with you at work; or this leader joked around with you at work.”) or in a serious manner at work (“Please recall a particular incident in which the leader communicated with you in a serious, rigid manner at work within the last 3 months. This leader expressed seriousness with you at work. For example, this leader shared serious events or stories with you at work; this leader spread seriousness into many types of situations when interacting with you at work; or this leader did not joke around with you at work.”). The participants were randomly assigned to one of the two conditions. If they could recall such an incident, they were asked to provide the initials of the supervisor and to describe the incident in as much detail as possible. After completing the recall task, the respondents rated their perceived organizational support, work engagement, and job crafting intentions in the form of seeking resources, seeking challenges, reducing demands, and perceived leader humor in relation to the humorous episodes. The participants then provided information regarding their demographic variables. We excluded five participants because they failed to recall such a described incident (see Appendix for a detailed flow of the experimental procedure). The final sample included a total of 210 participants divided between the low-humor condition (*N* = 106) and the high-humor condition (*N* = 104).

### Measures

All of the surveys were conducted in Mandarin Chinese, and the English scales were translated into Mandarin Chinese following Brislin’s translation/back-translation procedure (Brislin, 1970). We slightly adapted the items in Study 1 to fit the experimental scenario. After the participants recalled the incident, they were asked to provide the initials of the supervisor and rate the focal supervisor’s humor behaviors and their perceived organizational support, work engagement, and job crafting intention when working for this supervisor.

#### Leader Humor

We used the three-item leader humor scale (α = 0.96) developed and validated by Cooper et al. (2018). The answers were provided on a seven-point scale ranging from 1 = not at all to 7 = very much so. An example item is “This manager expressed humor with me at work.”

#### Perceived Organizational Support

Perceived organizational support (α = 0.91) was measured using a nine-item scale (Eisenberger et al., 1986). The following is a sample item: “My organization cared about my well-being.” All of the items were rated using a seven-point Likert scale ranging from 1 = strongly disagree to 7 = strongly agree.

#### Work Engagement

Work engagement (α = 0.95) was assessed with the nine-item shortened version of the Utrecht Work Engagement Scale (UWES; Schaufeli et al., 2006). The scale measures vigor with three items (e.g., “At my job, I will feel strong and vigorous”), dedication with three items (e.g., “I will be enthusiastic about my job”), and absorption with three items (e.g., “I will be immersed in my work”). All of the items were rated using a seven-point Likert scale ranging from 1 = strongly disagree to 7 = strongly agree.

#### Job Crafting Intention

We measured employee job crafting intention with the 13-item job crafting scale developed by Petrou et al. (2012), which consists of three dimensions. Seeking resources included six items (α = 0.83; e.g., “I will probably ask this supervisor for advice”); seeking challenges included three items (α = 0.88; e.g., “I will probably ask for more responsibilities”); and reducing demands included four items (α = 0.78; e.g., “I will probably attempt to ensure that my work is emotionally less intense”). All of the items were rated using a seven-point Likert scale ranging from 1 = almost never to 7 = very often.

#### Control Variables

We controlled for demographic variables, including sex, age, and tenure, because previous studies found that these variables could influence job crafting (Hetland et al., 2018).

#### Manipulation Checks

The effectiveness of the manipulations was verified. First, the efficacy of the leader humor behavior manipulations was measured using three items (e.g., “This manager expresses humor with me at work” [1 = almost never to 7 = very often]). The independent samples *t*-test indicated that the participants under the high-humor condition (*M* = 5.84, standard deviation (*SD*) = 0.77) were significantly more inclined to experience leader humor than those under the low-humor condition (*M* = 2.54, *SD* = 1.27, *t*(208) = −22.64, *p* < 0.001, Cohen’s *d* = −3.14). In addition, three independent undergraduate students who were blinded to the study’s hypotheses were asked to code the followers’ descriptions of leader humor behavior based on an overall judgment of their leader’s humor on a scale of 1 (extremely not humorous) to 7 (extremely humorous). An independent samples *t*-test revealed that leader humor was rated significantly higher under the high leader humor condition (*M* = 5.52, *SD* = 0.47) than under the low leader humor condition (*M* = 2.49, *SD* = 0.51, *t*(208) = −44.91, *p* < 0.001, Cohen’s *d* = −6.18). Thus, our manipulations of leader humor behavior were successful.

Similarly, our analysis showed that followers’ work engagement under the high-humor condition (*M* = 5.38, *SD* = 0.96) was rated significantly higher than that under the low-humor condition (*M* = 3.48, *SD* = 1.31, *t*(208) = −11.98, *p* < 0.001, Cohen’s *d* = −1.65), and followers’ perceived organizational support under the high-humor condition (*M* = 4.68, *SD* = 0.77) was rated significantly higher than that under the low-humor condition (*M* = 4.31, *SD* = 1.05, *t*(208) = −2.94, *p* < 0.01, Cohen’s *d* = −0.40). Moreover, followers’ seeking of resources under the high-humor condition (*M* = 5.61, *SD* = 1.01) was significantly higher than that under the low-humor condition (*M* = 5.28, *SD* = 0.82, *t*(208) = −3.00, *p* < 0.01, Cohen’s *d* = −0.36). Followers’ seeking of challenges under the high-humor condition (*M* = 5.18, *SD* = 0.97) was significantly higher than that under the low-humor condition (*M* = 4.01, *SD* = 1.51, *t*(208) = −6.67, *p* < 0.001, Cohen’s *d* = −0.92). Followers’ reducing of demands under the high-humor condition (*M* = 5.18, *SD* = 0.94) was significantly higher than that under the low-humor condition (*M* = 4.56, *SD* = 1.17, *t*(208) = −4.27, *p* < 0.001, Cohen’s *d* = −0.58).

Table 1 displays the means and standard deviations of the dependent variables by experimental condition (i.e., high leader humor and low leader humor).

**TABLE 1:** Study 1 means and standard deviations of the dependent variables by experimental condition.

Dependent variable	Low-humor condition		High-humor condition	
	*N* = 106		*N* = 104	
	*M*	*SD*	*M*	*SD*
Leader humor	2.54	1.27	5.84	0.77
Work engagement	3.48	1.31	5.38	0.96
Organizational support	4.31	1.05	4.68	0.77
Seeking resources	5.23	1.01	5.61	0.82
Reducing demands	4.56	1.17	5.18	0.94
Seeking challenges	4.01	1.51	5.18	0.97

We tested the hypothesized model to analyze the direct effects of leader humor on follower work engagement (Hypothesis 1), the direct effect of follower perceived organizational support on work engagement (Hypothesis 2), and the direct effects of follower perceived organizational support in the form of seeking resources (Hypothesis 3a), seeking challenges (Hypothesis 3b), and reducing demands (Hypothesis 3c). The serial mediating effects of leader humor on employee job crafting *via* work engagement and perceived organizational support (Hypothesis 4) were estimated, and ordinary least squares regression was used to test Hypotheses 1–3. Furthermore, a bootstrapping-based mediation test using the PROCESS macro (Hayes, 2013) was conducted to test Hypothesis 4. Following the suggestions of Preacher and Hayes (2008), we used unstandardized coefficients and a bootstrapping procedure to produce a 95% confidence interval (CI) around the estimated indirect effects. Accordingly, if the bias-corrected 95% CI excluded zero, the bootstrapped indirect effect was regarded as significant.

### Mediator Model

**FIGURE 2:**
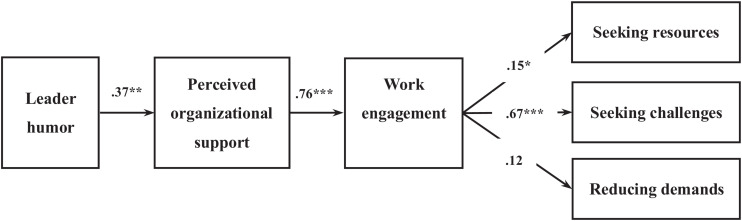
Study 1 sequential mediating model of the effect of leader humor on followers’ job crafting through followers’ POS and work engagement.

The unstandardized coefficients are presented herein. In accordance with Hypothesis 1, leader humor correlates positively with organizational support as perceived by employees (*b* = 0.37, standard error (*SE*) = 0.13, CI = [0.12, 0.62]). As predicted by Hypothesis 2, perceived organizational support is positively associated with work engagement (*b* = 0.76, *SE* = 0.07, CI = [0.00, 0.63]). With respect to Hypothesis 3, work engagement is associated with an increase in seeking resources (*b* = 0.15, *SE* = 0.06, CI = [0.02, 0.27]) and seeking challenges (*b* = 0.67, *SE* = 0.07, CI = [0.52, 0.81]) but not associated with reducing demands (*b* = 0.12, *SE* = 0.08, CI = [−0.04, 0.29]). These results partially support Hypothesis 3. As proposed in Hypothesis 4, employee-perceived organizational support and work engagement sequentially mediate the positive link between leader humor and employee job crafting in the form of seeking resources (*b* = 0.04, *SE* = 0.02, CI = [0.01, 0.10]) and seeking challenges (*b* = 0.19, *SE* = 0.07, CI = [0.07, 0.34]) but not in the form of reducing demands (*b* = 0.04, *SE* = 0.03, CI = [−0.01, 0.11]). Thus, Hypothesis 4 is partially supported. Accordingly, leader humor is linked to perceived organizational support, which is related to better employee work engagement and subsequently leads to a higher level of employee job crafting in the form of seeking resources and challenges. These effects persist regardless of the inclusion or exclusion of the control variables. Table 2 and Figure 2 presents the estimates of the path coefficients, the indirect effects, and the bias-corrected 95% CIs.

**TABLE 2:** Study 1 path coefficients and indirect effects in the multiple mediation model.

Model	Path coefficients					Indirect effects on SR		Indirect effects on RD		Indirect effects on SC	
	To POS	To WE	To SR	To RD	To SC	Estimate	95% CI	Estimate	95% CI	Estimate	95% CI
LH	0.37 (0.13)	1.60 (0.12)	−0.05 (0.15)	0.29 (0.19)	−0.19 (0.17)						
POS		0.76 (0.07)	0.42 (0.08)	0.26 (0.10)	0.24 (0.09)						
WE			0.15 (0.06)	0.12 (0.08)	0.67 (0.07)						
Total						0.43 (0.12)	0.21, 0.69	0.33 (0.16)	0.03, 0.64	1.35 (0.19)	0.99, 1.72
X-M1-Y						0.16 (0.07)	0.05, 0.32	0.10 (0.05)	0.02, 0.24	0.09 (0.05)	0.01, 0.23
X-M1-M2-Y						0.04 (0.02)	0.01, 0.10	0.04 (0.03)	−0.01, 0.11	0.19 (0.07)	0.07, 0.34
X-M2-Y						0.23 (0.10)	0.05, 0.43	0.20 (0.14)	−0.07, 0.48	1.07 (0.17)	0.74, 1.41

*LH, leader humor; WE, work engagement; POS, perceived organizational support; SR, seeking resources; M2, work engagement as a mediator; M1, perceived organizational support; RD, reducing demands; SC, seeking challenges; CI, confidence interval.*

## Study 2

### Sample and Procedures

The participants were recruited from a consulting company in central China. To ensure authenticity, we restricted the participants to those with daily direct communication with their immediate manager (Wheeler et al., 2014). According to theoretical research by Podsakoff et al. (2012), temporal separation is one of the most effective means of reducing common method variance (CMV) since temporal separation allows previously recalled information to leave short-term memory. Thus, consistent with previous empirical studies (e.g., Wang et al., 2018; Yam et al., 2018), we chose a 2-week interval to reduce CMV bias while avoiding missing data caused by the time lag. At time 1, leader humor and employees’ sex, age, and education were measured, and 527 valid questionnaires were retrieved. At time 2, employee work engagement, perceived organizational support, and job crafting in the form of seeking resources, seeking challenges, and reducing demands, respectively, were measured. We disqualified responses that were determined to be untrue or that demonstrated inconsistent logic by using one attention check item (“Please select strongly agree for this question”). After accounting for missing data, 406 valid questionnaires were retrieved, rendering a response rate of 63.54%. Of the 406 participants, 156 were female (38.4%), and 250 were male (61.6%). More than half of participants were aged between 30 and 39 years old (55.1%), 13.1% were between 20 and 29 years old, 24.4% were between 40 and 49 years old, and 7.4% were older than the age of 50. The average for organizational tenure was 8.4 years.

### Measures

#### Leader Humor

We used the three-item leader humor scale (α = 0.83) developed and validated by Cooper et al. (2018). The answers were provided on a seven-point scale ranging from 1 (not at all) to 7 (very much so). The items are: “This manager expresses humor with me at work.”, “I’ve seen my manager inject humor into many types of situations when interacting with me.”, and “My manager jokes around with me.”

Perceived organizational support (α = 0.89) and job crafting behavior in the forms of seeking resources (α = 0.70), seeking challenges (α = 0.72), and reducing demands (α = 0.66) were measured using the same items described in Study 1.

We assessed work engagement (α = 0.90) using the original 17-item UWES (Schaufeli et al., 2002; Salanova and Schaufeli, 2008). The scale measured vigor with six items (e.g., “When I get up in the morning, I feel like going to work”), dedication with five items (e.g., “I am enthusiastic about my job”), and absorption with six items (e.g., “When I am working, I forget everything else around me”). Because prior studies have found that demographic variables could be positively correlated with employees’ proactive behaviors (Zhang et al., 2012; Braun and Nieberle, 2017), we controlled for the employees’ demographic variables, including gender, age, and subordinate-reported dyadic tenure (Hetland et al., 2018).

### Correlation Analyses

As proposed, the results replicated those found in Study 1. Table 3 displays the descriptive statistics of all of the study variables in Study 2.

**TABLE 3:** Study 2 means, standard deviations, and the correlation matrix of variables (*N* = 406).

	*M*	*SD*	1	2	3	4	5	6	7	8	9
(1) Leader humor	4.09	1.07	–								
(2) Seeking resources	5.10	0.68	0.28**	–							
(3) Reducing demands	5.15	0.80	0.29**	0.57**	–						
(4) Seeking challenges	5.37	0.88	0.26**	0.53**	0.44**	–					
(5) Work engagement	4.32	0.75	0.31**	0.40**	0.37**	0.41**	–				
(6) Organizational support	4.86	0.94	0.47**	0.41**	0.37**	0.46**	0.53**	–			
(7) Sex	0.38	0.49	−0.02	0.02	0.01	−0.09	−0.09	−0.06	–		
(8) Tenure	8.44	5.46	−0.07	−0.03	−0.09	0.06	0.13*	0.02	−0.21**	–	
(9) Age	2.26	0.78	−0.03	−0.02	−0.09	0.03	0.15**	0.02	−0.18**	0.71**	–

*p < 0.05 and **p < 0.01.

As shown in Table 4 we conducted a series of confirmatory factor analyses (CFAs) to examine the distinctiveness of our six key variables using Mplus software (Muthén and Muthén, 2010). We used item parcels to reduce the number of indicators to 3 for each dimension of work engagement (Little et al., 2002). The results of the CFA demonstrated that the hypothesized six-factor model, consisting of leader humor, perceived organizational support, work engagement, seeking resources, seeking challenges, and reducing demands, fit the data reasonably: *χ*^2^ (309) = 608.22, root mean square error of approximation (RMSEA) = 0.049, comparative fit index (CFI) = 0.931. This model was superior over alternative models, including a five-factor model in which the organizational support perceived by followers and work engagement were constrained to a single factor (Δ*χ*^2^ (5) = 341.88, *p* < 0.01, RMSEA = 0.071, CFI = 0.853); a four-factor model in which leader humor, organizational support perceived by employees, and work engagement were constrained to a single factor (Δ*χ*^2^ (9) = 748.12, *p* < 0.01, RMSEA = 0.090, CFI = 0.760); a three-factor model in which leader humor, perceived organizational support, work engagement, and seeking resources were constrained to one factor (Δ*χ*^2^ (12) = 1055.23, *p* < 0.01, RMSEA = 0.101, CFI = 0.088); a two-factor model in which leader humor, perceived organizational support, work engagement, seeking resources, and seeking challenges were constrained to one factor (Δ*χ*^2^ (14) = 1225.5, *p* < 0.01, RMSEA = 0.107, CFI = 0.651); and a one-factor model in which all variables were set to load onto a single factor (Δ*χ*^2^ (15) = 1286.77, *p* < 0.01, RMSEA = 0.109, CFI = 0.637).

**TABLE 4:** Study 2 fit indices of alternative models.

Model^*a*^	*χ*^2^	df	CFI	TLI	RMSEA	SRMR	*χ*^2^ difference (df)^*b*^
Six factors (intended)^*c*^	608.22	309	0.931	0.921	0.049	0.052	–
Five factors^*d*^	950.10	314	0.853	0.836	0.071	0.063	341.88 (5)**
Four factors^*e*^	1356.34	318	0.760	0.735	0.090	0.072	748.12 (9)**
Three factors^*f*^	1663.45	321	0.690	0.661	0.101	0.088	1055.23 (12)**
Two factors^*g*^	1833.72	323	0.651	0.621	0.107	0.091	1225.5 (14)**
One factor^*h*^	1894.99	324	0.637	0.607	0.109	0.091	1286.77 (15)**

df, degree of freedom; CFI, comparative fit index; TLI, Tucker–Lewis index; RMSEA, root mean square error of approximation; SRMR, standardized root mean square residual. **p < 0.01. ^*a*^Baseline model: *χ*^2^ = 7604.37, df = 861. ^*b*^Comparison to the intended measurement model with six factors. ^*c*^Intended measurement model. ^*d*^Model with five factors: (1) leader humor, (2) perceived organizational support + work engagement, (3) seeking resources, (4) seeking challenges, and (5) reducing demands. ^*e*^Model with four factors: (1) leader humor + perceived organizational support + work engagement, (2) seeking resources, (3) seeking challenges, and (4) reducing demands. ^*f*^Model with three factors: (1) leader humor + perceived organizational support + work engagement + seeking resources, (2) seeking challenges, and (3) reducing demands. ^*g*^Model with two factors: (1) leader humor + perceived organizational support + work engagement + seeking resources + seeking challenges and (2) reducing demands. ^*h*^Model with one factor: All items combined into one factor.

### Mediator Model

**FIGURE 3:**
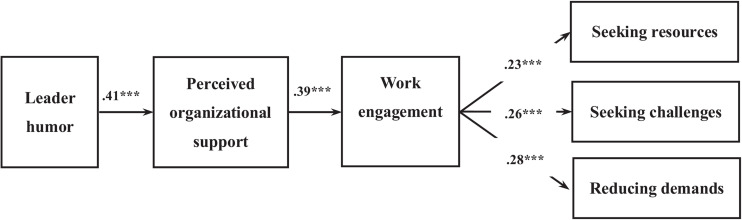
Study 2 sequential mediating model of the effect of leader humor on followers’ job crafting through followers’ POS and work engagement.

The unstandardized coefficients are reported herein. The results of Study 1 are consistent with those of Study 2. In support of Hypothesis 1, leader humor is positively associated with organizational support perceived by employees (*b* = 0.41, *SE* = 0.04, CI = [0.33, 0.49]). As proposed in Hypothesis 2, perceived organizational support is positively correlated with work engagement (*b* = 0.39, *SE* = 0.04, CI = [0.31, 0.46]). With respect to Hypothesis 3, employee work engagement is positively associated with employees seeking resources behavior (*b* = 0.23, *SE* = 0.05, CI = [0.13, 0.32]) and seeking challenges behavior (*b* = 0.26, *SE* = 0.06, CI = [0.14, 0.38]). Moreover, employee work engagement is unexpectedly positively associated with reducing demands (*b* = 0.28, *SE* = 0.06, CI = [0.16, 0.39]). In accordance with Hypothesis 4, employee-perceived organizational support and work engagement sequentially mediate the positive link between leader humor and employee job crafting in the form of seeking resources (*b* = 0.04, *SE* = 0.01, CI = [0.02, 0.06]), seeking challenges (*b* = 0.04, *SE* = 0.01, CI = [0.02, 0.07]), and reducing demands (*b* = 0.04, *SE* = 0.01, CI = [0.02, 0.07]). Hence, leader humor is positively linked to perceived organizational support, which is related to an increase in employee work engagement, and subsequently, it relates to a high level of employee job crafting. Table 5 and Figure 3 presents the estimates of the path coefficients, the indirect effects, and the bias-corrected 95% CIs.

**TABLE 5:** Study 2 path coefficients and indirect effects in the multiple mediation model.

Model	Path coefficients					Indirect effects on SR		Indirect effects on RD		Indirect effects on SC	
	To POS	To WE	To SR	To RD	To SC	Estimate	95% CI	Estimate	95% CI	Estimate	95% CI
LH	0.41 (0.04)	0.06 (0.03)	0.06 (0.03)	0.09 (0.04)	0.03 (0.04)						
POS		0.39 (0.04)	0.17 (0.04)	0.15 (0.05)	0.30 (0.05)						
WE			0.23 (0.05)	0.28 (0.06)	0.26 (0.06)						
Total						0.12 (0.02)	0.08, 0.16	0.12 (0.03)	0.08, 0.18	0.17 (0.03)	0.13, 0.24
X-M1-Y						0.07 (0.02)	0.03, 0.11	0.06 (0.02)	0.02, 0.11	0.12 (0.03)	0.07, 0.18
X-M1-M2-Y						0.04 (0.01)	0.02, 0.06	0.04 (0.01)	0.02, 0.07	0.04 (0.01)	0.02, 0.07
X-M2-Y						0.01 (0.01)	−0.00, 0.03	0.02 (0.01)	0.00, 0.04	0.02 (0.01)	−0.00, 0.04

*LH, leader humor; WE, work engagement; POS, perceived organizational support; SR, seeking resources; M2, work engagement as a mediator; M1, perceived organizational support; RD, reducing demands; SC, seeking challenges; CI, confidence interval.*

## Discussion

In summary, by integrating an experimental design and a field survey study, we find the proposed indirect links between leader humor and employee job crafting behaviors in the form of seeking resources, seeking challenges, and reducing demands through employee work engagement and perceived organizational support. The theoretical and managerial implications of our findings are discussed below.

### Theoretical Implications

Our study extends the theory and research related to leader humor and job crafting in several ways. First, we contribute to the leader humor literature and the job crafting literature by focusing on the positive consequences of leader humor behaviors on job crafting by followers. Our results provide evidence that leader humor can facilitate follower job crafting. Job crafting is the focus of a new and growing body of literature that demonstrates a variety of gains caused by such behavior, such as positive emotions, satisfaction, and person-job fit (Demerouti et al., 2015; Tims et al., 2016; Van Wingerden et al., 2017; Bindl et al., 2018; Bruning and Campion, 2018; Gordon et al., 2018). By viewing leader humor through the lens of the JD-R model, our study explores the factors that facilitate followers’ job crafting behavior (Li et al., 2013), and it answers calls from scholars to examine the positive effects of leader humor (Cooper, 2008).

Second, this study considers leader behavior when examining how to manage and promote employee work engagement and perceived organizational support, thereby contributing to the development of the work engagement and organizational support literature. Our study reveals that leader humor is positively related to employee-perceived organizational support and work engagement. Regarding the relationship between leader humor and followers’ perceived organizational support, we found a significant, direct link. More precisely, employees under the supervision of humorous leaders are more likely to believe that their organization values their contributions, cares about their well-being, and fulfills their needs. These results support the JD-R model, which posits that job resources (i.e., perceived organizational support) engendered by work environments, events, and behaviors impact work engagement (Schaufeli and Bakker, 2004). Our results reveal a significant relationship between leader humor and work engagement, consistent with prior studies that have discovered a positive link between leader sense of humor and employee work engagement (Yam et al., 2018).

Third, we extend the literature by uncovering the roles of employee-perceived organizational support and work engagement in mediating the association between leader humor and job crafting, thus responding to the call for studies to document the underlying mechanisms that mediate the relationships between leader behaviors and job crafting (Berg et al., 2013; Wang et al., 2017). The results support the notion that perceived organizational support and work engagement serially mediate the link between leader humor and job crafting. Our result is also consistent with the JD-R model, which posits that work engagement mediates the associations between job resources and employee outcomes (Schaufeli et al., 2009). Thus, when working with a humorous leader, followers tend to have high levels of perceived organizational support and are subsequently more likely to be engaged in their work and in crafting their jobs. More specifically, seeking resources and seeking challenges, which are expansion strategies of behavioral job crafting (Petrou et al., 2012; Costantini et al., 2019), might be more positively related to employee work engagement. Unexpectedly, in contradiction with our hypothesis, our findings in this study demonstrate that reducing demands appears to be unrelated to, or positively associated with, employee work engagement. This result is contradicted by a majority of studies that have suggested that reducing demands, as a contraction strategy of behavioral job crafting, is negatively related to work engagement. The existing literature has also pointed to some possible negative consequences of job crafting. For example, the literature on job crafting suggests that decreasing hindering demands leads to negative work-related well-being outcomes (Rudolph et al., 2017; Zhang and Parker, 2019). However, Costantini et al. (2019) suggested that individuals not only minimize demands (i.e., make work less intense) but also optimize demands (i.e., make work more efficient) to restore the fit between individuals’ demands and preferences. Hence, future studies could include both minimizing demands and optimizing demands to gain a more comprehensive understanding of job crafting.

### Managerial Implications

Our study suggests that leaders should consider using humor to motivate employees to craft their jobs. For example, leaders could provide individualized support to build a trusting, open, and supportive climate in which job crafting is encouraged. Leaders could also display behaviors signaling openness and support, such as listening to employees’ individual needs (Decker and Rotondo, 2001). As a result, employees might feel free and safe to craft their job demands and job resources. The findings of the mediation effect suggest that an effective way to increase job crafting in the form of seeking resources and addressing challenges is by improving employee work engagement and perceived organizational support. Therefore, organizations might consider increasing employee work engagement and perceived organizational support to promote their job crafting. Developing leader humor is one approach to achieving this goal. However, our results revealed that leader humor enables employees to reduce demands by increasing their perceived organizational support and work engagement.

Our theoretical model and empirical findings also have critical practical implications for practitioners and human resource management in organizations. Generally, our results indicate that humorous leaders can enhance employee proactive behaviors at work in the form of job crafting. Moreover, the organizational support perceived by employees and employee work engagement serially mediate the positive relationship between leader humor and the job crafting behaviors of employees. These findings shed light on the salient and important role of leader humor in fostering employees’ proactive behaviors. Specifically, having fun and using humor at work are effective and essential ways to improve the proactivity of employees’ work lives, such as job crafting. Leader humor has received increasing attention in organizations due to its significant benefits, such as strengthening followers’ job performance and mental health and improving organizational effectiveness (Mesmer-magnus et al., 2012).

### Limitations and Future Research Directions

First, the use of self-reports could raise concern regarding CMV bias (Podsakoff et al., 2012), but this concern is reduced for a few reasons. First, CMV is reduced due to the 2-week interval between times 1 and 2. While the variables in Study 1 were measured at the same time that the mediators and outcomes from the same time point in Study 2 were tested, the predictor was temporally separated from the mediators in Study 2. Temporal separation, which is as effective as source separation, is among the most effective means for reducing CMV (Podsakoff et al., 2012). Moreover, in the current study, we aimed to test how leader humor is associated with employee work engagement, perceived organizational support, and self-initiated behavior (e.g., job crafting). According to the JD-R model, each job and each individual have his or her own group of job demands and resources. Hence, employees are a good source of information concerning their own crafting behaviors. Additionally, we tested the relationship between leader humor and employee work engagement/perceived organizational support, which is a personal experience/perception. Finally, we applied Podsakoff et al.’ (2012) method to diminish the impact of common method bias by ensuring participant anonymity. Overall, it seems that CMV is less likely to be a major concern in our study.

Second, another limitation is related to causality. It is difficult to draw causal conclusions based on field and survey studies. However, we adopted measures to strengthen the nature of causality in the current study. We assessed our dependent variables separately from our independent variables, which prior studies have argued enhances causal inference (Podsakoff et al., 2012).

Third, future research could examine potential boundary conditions. Consistent with the JD-R model, job strain might moderate the indirect link of resources (work engagement and perceived organizational support) between leader humor and job crafting behavior (Halbesleben et al., 2014). When followers experience a higher level of job strain, the resources, i.e., perceived organizational support, engendered by leader humor are less likely to translate into job crafting behaviors through work engagement. Moreover, in line with the notion that behaviors and intentions are generally linked, we measured job crafting intentions and behaviors in Studies 1 and 2, respectively, and found that the results of these two studies were consistent with one another. However, we acknowledge that intentions do not always translate into behaviors. Thus, our results should be further validated in a future study.

## Conclusion

Building upon JD-R theory, we conclude that, as a socioemotional resource, leader humor is a salient facilitator of followers’ proactive behaviors in the form of job crafting behavior. More specifically, leader humor can enhance employees’ perceived organizational support and work engagement and, in turn, strengthen employees’ tendency to craft their jobs.

## Data Availability Statement

The datasets generated for this study are available on request to the corresponding author.

## Ethics Statement

The study was conducted in accordance with the guidelines established by the Declaration of Helsinki and in compliance with the APA ethical guidelines. The patients/participants provided their written informed consent to participate in this study.

## Author Contributions

LT: data collection, data cleaning, theory building, writing, and data analysis. YW and HL: theory building and data collection. WQ: data collection, cleaning and editing, and figures. All authors contributed to the article and approved the submitted version.

## Conflict of Interest

The authors declare that the research was conducted in the absence of any commercial or financial relationships that could be construed as a potential conflict of interest.

## Footnotes

1 https://www.wjx.cn

## Appendix

Flow of procedures and participant instructions in Study 1.

## References

[B1] Arendt L. A. (2009). Transformational leadership and follower creativity: the moderating effect of leader humor. *Rev. Bus. Res.* 9 100–106.

[B2] Avolio B. J. Howell J. M. Sosik J. J. (1999). A funny thing happened on the way to the bottom line: Humor as a moderator of leadership style effects. *Acad. Manag. J.* 42 219–227. 10.2307/257094

[B3] Bakker A. B. Tims M. Derks D. (2012). Proactive personality and job performance: The role of job crafting and work engagement. *Hum. Relat.* 65 1359–1378. 10.1177/0018726712453471

[B4] Berg J. M. Dutton J. E. Wrzesniewski A. (2013). “Job crafting and meaningful work,” in *Purpose and meaning in the workplace*, eds Dik B. J. Byrne Z. S. Steger M. F., (Washington: American Psychological Association), 81–104. 10.1037/14183-005

[B5] Bindl U. K. Hardin K. Gibson C. B. Stride C. B. (2018). Job crafting revisited: Implications of an extended framework for active changes at work. *J. Appl. Psychol.* 104 605–628. 10.1037/apl0000362 30407042

[B6] Bipp T. Demerouti E. (2015). Which employees craft their jobs and how? Basic dimensions of personality and employees’ job crafting behaviour. *J. Occupat. Organizat. Psychol.* 88 631–655. 10.1111/joop.12089

[B7] Bitterly T. B. Schweitzer M. E. (2019). The impression management benefits of humorous self-disclosures: how humor influences perceptions of veracity. *Organizat. Behav. Hum. Decis. Proc.* 151 73–89. 10.1016/j.obhdp.2019.01.005

[B8] Braun S. Nieberle K. W. A. M. (2017). Authentic leadership extends beyond work: A multilevel model of work-family conflict and enrichment. *Leadersh. Q.* 28 780–797. 10.1016/j.leaqua.2017.04.003

[B9] Brislin R. W. (1970). Back-translation for cross-cultural research. *J. Cross Cult. Psychol.* 1 185–216. 10.1177/135910457000100301

[B10] Bruning P. F. Campion M. A. (2018). A Role-resource Approach-avoidance model of job crafting: A multimethod integration and extension of job crafting theory. *Acad. Manag. J.* 61 499–522. 10.5465/amj.2015.0604

[B11] Caesens G. Marique G. Hanin D. Stinglhamber F. (2016). The relationship between perceived organizational support and proactive behaviour directed towards the organization. *Eur. J. Work Organizat. Psychol.* 25 398–411. 10.1080/1359432x.2015.1092960

[B12] Caesens G. Stinglhamber F. Luypaert G. (2014). The impact of work engagement and workaholism on well-being-the role of work-related social support. *Career Develop. Int.* 19 813–835. 10.1108/cdi-09-2013-0114

[B13] Christian M. S. Garza A. S. Slaughter J. E. (2011). Work engagement: A quantitative review and test of its relations with task and contextual performance. *Person. Psychol.* 64 89–136. 10.1111/j.1744-6570.2010.01203.x

[B14] Cooper C. (2008). Elucidating the bonds of workplace humor: A relational process model. *Hum. Relat.* 61 1087–1115. 10.1177/0018726708094861

[B15] Cooper C. D. Kong D. T. Crossley C. D. (2018). Leader humor as an interpersonal resource:Intergrating three theoretical perspective. *Acad. Manag. J.* 61 769–796. 10.5465/amj.2014.0358

[B16] Costantini A. Demerouti E. Ceschi A. Sartori R. (2019). Evidence on the hierarchical, multidimensional nature of behavioural job crafting. *Appl. Psychol.* 0 1–31. 10.1111/apps.12232

[B17] Decker W. H. Rotondo D. M. (2001). Relationships among gender, type of humor, and perceived leader effectiveness. *J. Manag. Issues* 13 450–465.

[B18] Demerouti E. Bakker A. B. Gevers J. M. P. (2015). Job crafting and extra-role behavior: the role of work engagement and flourishing. *J. Vocat. Behav.* 91 87–96. 10.1016/j.jvb.2015.09.001

[B19] Demerouti E. Bakker A. B. Nachreiner F. Schaufeli W. B. (2001). The job demands-resources model of burnout. *J. Appl. Psychol.* 86 499–512. 10.1037/0021-9010.86.3.499 11419809

[B20] Den Hartog D. N. Belschak F. D. (2012). Work engagement and machiavellianism in the ethical leadership process. *J. Bus. Ethics* 107 35–47. 10.1007/s10551-012-1296-4

[B21] Duncan W. J. (1982). Humor in management: Prospects for administrative practice and research. *Acad. Manag. Rev.* 7 136–142. 10.5465/amr.1982.4285511

[B22] Eisenberger R. Huntington R. Hutchison S. O. Sowa D. (1986). Perceived Organisational Support. *J. Appl. Psychol.* 71 500–507.

[B23] Eisenberger R. Stinglhamber F. (2011). *Perceived Organizational Support: Fostering Enthusiastic and Productive Employees*. Washington, DC: American Psychological Association.

[B24] Flanagan J. C. (1954). The critical incident technique. *Psychol. Bull.* 51 327–358. 13177800 10.1037/h0061470

[B25] Gordon H. J. Demerouti E. Le Blanc P. M. Bakker A. B. Bipp T. Verhagen M. A. M. T. (2018). Individual job redesign: Job crafting interventions in healthcare. *J. Vocat. Behav.* 104 98–114. 10.1016/j.jvb.2017.07.002

[B26] Gremler D. D. (2015). “The Critical Incident Technique,” in *Wiley Encyclopedia of Management*, eds Edn, eds Cooper C. L. Lee N. Farrell A. M., (New Jersey: Wiley). 10.1002/9781118785317.weom090062

[B27] Halbesleben J. R. B. Neveu J. P. Paustian-Underdahl S. C. Westman M. (2014). Getting to the “COR”: Understanding the role of resources in conservation of resources theory. *J. Manag.* 40 1334–1364. 10.1177/0149206314527130

[B28] Hallberg U. E. Schaufeli W. B. (2006). ”Same Same” but different? But Can work engagement be discriminated from job involvement and organizational commitment? *J. Europ. Psychol.* 11 119–127. 10.1027/1016-9040.11.2.119

[B29] Hayes A. F. (2013). *An Introduction to Mediation, Moderation, and Conditional Process Analysis: A Regression-Based Approach.* New York: Guilford Press.

[B30] Hetland J. Hetland H. Bakker A. B. Demerouti E. (2018). Daily transformational leadership and employee job crafting: The role of promotion focus. *Eur. Manag. J.* 36 746–756. 10.1016/j.emj.2018.01.002

[B31] Huang L. Gino F. Galinsky A. D. (2015). The highest form of intelligence: sarcasm increases creativity for both expressers and recipients. *Organ. Behav. Human Decision Process.* 131 162–177. 10.1016/j.obhdp.2015.07.001

[B32] Hughes L. W. Avey J. B. (2009). Transforming with levity: humor, leadership, and follower attitudes. *Lead. Organ. Dev. J.* 30 540–562. 10.1108/01437730910981926

[B33] Ju D. Huang M. Liu D. Qin X. Hu Q. Chen C. (2019). Supervisory consequences of abusive supervision: an investigation of sense of power, managerial self-efficacy, and task-oriented leadership behavior. *Organizat. Behav. Hum. Decis. Proc.* 154 80–95. 10.1016/j.obhdp.2019.09.003

[B72] Kahn W. A. (1990). Psychological conditions of personal engagement and disengagement at work. *Acad. Manag. J.* 33, 692–724. 10.5465/256287

[B34] Kong D. T. Cooper C. D. Sosik J. J. (2019). The state of research on leader humor. *Organ. Psychol. Rev.* 9 3–40. 10.1177/2041386619846948

[B35] Kurtessis J. N. Eisenberger R. Ford M. T. Buffardi L. C. Stewart K. A. Adis C. (2017). Perceived Organizational Support A Meta-Analytic Evaluation of Organizational Support Theory. *J. Manag.* 43 1854–1884. 10.1177/0149206315575554

[B36] Li N. Chiaburu D. S. Kirkman B. L. Xie Z. (2013). Spotlight on the followers: An examination of moderators of relationships between transformational leadership and subordinates’ citizenship and taking charge. *Person. Psychol.* 66 225–260. 10.1111/peps.12014

[B37] Liang L. H. Lian H. Brown D. J. Ferris D. L. Hanig S. Keeping L. M. (2016). Why are abusive supervisors abusive? A dual-system self-control model. *Acad. Manag. J.* 59 1385–1406. 10.5465/amj.2014.0651

[B38] Lichtenthaler P. W. Fischbach A. (2019). A meta-analysis on promotion- and prevention-focused job crafting. *Eur. J. Work Organizat. Psychol.* 28(1), 30–50. 10.1080/1359432x.2018.1527767

[B39] Little T. D. Cunningham W. A. Shahar G. Widaman K. F. (2002). To parcel or not to parcel: exploring the question, weighing the merits. *Struct. Equ. Model.* 9 151–173. 10.1207/S15328007SEM0902_1

[B40] May D. Gilson R. Harter L. (2004). The psychological conditions of meaningfulness, safety and availability and the engagement of the human spirit at work. *J. Occup. Organ. Psychol.* 77 11–37. 10.1348/096317904322915892 30467716

[B41] McGraw A. P. Warren C. (2010). Benign violations: Making immoral behavior funny. *Psychol. Sci.* 21 1141–1149. 10.1177/0956797610376073 20587696

[B42] Mesmer-magnus J. Glew D. J. Viswesvaran C. (2012). A meta-analysis of positive humor in the workplace. *J. Manag. Psychol.* 27 155–190. 10.1108/02683941211199554

[B43] Muthén L. K. Muthén B. O. (2010). *MplusUser’s guide*, 6th Edn California: Los Angeles.

[B44] Parker S. K. Williams H. M. Turner N. (2006). Modeling the antecedents of proactive behavior at work. *J. Appl. Psychol.* 91 636–652. 10.1037/0021-9010.91.3.636 16737360

[B45] Petrou P. Demerouti E. Peeters M. C. W. Schaufeli W. B. Hetland J. (2012). Crafting a job on a daily basis: Contextual correlates and the link to work engagement. *J. Organiz. Behav.* 33 1120–1141. 10.1002/job.1783

[B46] Petrou P. Demerouti E. Schaufeli W. B. (2018). Crafting the change: The role of employee job crafting behaviors for successful organizational change. *J. Manag.* 44 1766–1792. 10.1177/0149206315624961

[B47] Podsakoff P. M. Mackenzie S. B. Podsakoff N. P. (2012). Sources of method bias in social science research and recommendations on how to control it. *Annu. Rev. Psychol.* 63:539. 10.1146/annurev-psych-120710-100452 21838546

[B48] Preacher K. J. Hayes A. F. (2008). “Contemporary Approaches to Assessing Mediation in Communication Research,” in *The Sage Sourcebook of Advanced Data Analysis Methods for Communication Research*, Eds Edn, eds Hayes A. F. Slater M. D. Synder L. (Thousand Oaks, CA: Sage), 13–54. 10.4135/9781452272054.n2

[B49] Pundt A. Venz L. (2017). Personal need for structure as a boundary condition for humor in leadership. *J. Organizat. Behav.* 38 87–107. 10.1002/job.2112

[B50] Rhoades L. Eisenberger R. (2002). Perceived organizational support: A review of the literature. *J. Appl. Psychol.* 87 698–714. 12184574 10.1037/0021-9010.87.4.698

[B51] Rich B. L. Lepine J. A. Crawford E. R. (2010). Job engagement: Antecedents and effects on job performance. *Acad. Manag. J.* 53 617–635. 10.5465/amj.2010.51468988

[B52] Robert C. Dunne T. C. Iun J. (2015). The impact of leader humor on subordinate job satisfaction. *Group Organizat. Manag.* 41 375–406. 10.1177/1059601115598719

[B53] Rudolph C. W. Katz I. M. Lavigne K. N. Zacher H. (2017). Job crafting: a meta-analysis of relationships with individual differences, job characteristics, and work outcomes. *J. Vocat. Behav.* 102 112–138. 10.1016/j.jvb.2017.05.008

[B54] Salanova M. Schaufeli W. B. (2008). A cross-national study of work engagement as a mediator between job resources and proactive behaviour. *Int. J. Hum. Resour. Manag.* 19 116–131. 10.1080/09585190701763982

[B55] Schaufeli W. B. Bakker A. B. (2004). Job demands, job resources, and their relationship with burnout and engagement: A multi-sample study. *J. Organizat. Behav.* 25 293–315. 10.1002/job.248

[B56] Schaufeli W. B. Bakker A. B. Salanova M. (2006). The measurement of work engagement with a short questionnaire-A cross-national study. *Educat. Psychol. Measur.* 66 701–716. 10.1177/0013164405282471

[B57] Schaufeli W. B. Bakker A. B. van Rhenen W. (2009). How changes in job demands and resources predict burnout, work engagement, and sickness absenteeism. *J. Organizat. Behav.* 30 893–917. 10.1002/job.595

[B73] Schaufeli W. B. Salanova M. González-Romá V. Bakker A. B. (2002). The measurement of engagement and burnout: a two sample confirmatory factor analytic approach. *J. Happin. Stud.* 3, 71–92. 10.1023/A:1015630930326

[B58] Sonnentag S. (2003). Recovery, work engagement, and proactive behavior: A new look at the interface between nonwork and work. *J. Appl. Psychol.* 88 518–528. 10.1037/0021-9010.88.3.518 12814299

[B59] Tims M. Bakker A. B. Derks D. (2012). Development and validation of the job crafting scale. *J. Vocat. Behav.* 80 173–186. 10.1016/j.jvb.2011.05.009

[B60] Tims M. Derks D. Bakker A. B. (2016). Job crafting and its relationships with person-job fit and meaningfulness: A three-wave study. *J. Vocat. Behav.* 92 44–53. 10.1016/j.jvb.2015.11.007

[B61] Tremblay M. Gibson M. (2015). The role of humor in the relationship between transactional leadership behavior, perceived supervisor support, and citizenship behavior. *J. Leadersh. Organizat. Stud.* 23 39–54. 10.1177/1548051815613018

[B62] Van Wingerden J. Derks D. Bakker A. B. (2017). The impact of personal resources and job crafting interventions on work engagement and performance. *Hum. Resour. Manag.* 56 51–67. 10.1002/hrm.21758

[B63] Vecchio R. P. Justin J. E. Pearce C. L. (2009). The influence of leader humor on relationships between leader behavior and follower outcomes. *J. Manag. Issu.* 21 171–194.

[B64] Vogelgesang G. R. Leroy H. Avolio B. J. (2013). The mediating effects of leader integrity with transparency in communication and work engagement/performance. *Lead. Quart.* 24 405–413. 10.1016/j.leaqua.2013.01.004

[B65] Wang H.-J. Demerouti E. Le Blanc P. (2017). Transformational leadership, adaptability, and job crafting: The moderating role of organizational identification. *J. Vocat. Behav.* 100 185–195. 10.1016/j.jvb.2017.03.009

[B66] Wang L. Restubog S. Shao B. Lu V. Van Kleef G. A. (2018). Does anger expression help or harm leader effectiveness? The role of competence-based versus integrity-based violations and abusive supervision. *Acad. Manag. J.* 61 1050–1072. 10.5465/amj.2015.0460

[B67] Wellman N. Mayer D. M. Ong M. Derue D. S. (2016). When are do-gooders treated badly? Legitimate power, role expectations, and reactions to moral objection in organizations. *J. Appl. Psychol.* 101 793–814. 10.1037/apl0000094 26882445

[B68] Wheeler A. R. Shanine K. K. Leon M. R. Whitman M. V. (2014). Student-recruited samples in organizational research: A review, analysis, and guidelines for future research. *J. Occupat. Organizat. Psychol.* 87 1–26. 10.1111/joop.12042

[B69] Yam K. C. Christian M. S. Wei W. Liao Z. Nai J. (2018). The mixed blessing of leader sense of humor: Examining costs and benefits. *Acad. Manag. J.* 61 348–369. 10.5465/amj.2015.1088

[B70] Zhang F. Parker S. K. (2019). Reorienting job crafting research: a hierarchical structure of job crafting concepts and integrative review. *J. Organizat. Behav.* 40 126–146. 10.1002/job.2332

[B71] Zhang H. Kwan H. K. Everett A. M. Jian Z. (2012). Servant leadership, organizational identification, and work-to-family enrichment: The moderating role of work climate for sharing family concerns. *Hum. Resour. Manag.* 51 747–767. 10.1002/hrm.21498

